# Npt2a as a target for treating hyperphosphatemia

**DOI:** 10.1042/BST20211005

**Published:** 2022-01-07

**Authors:** Linto Thomas, Jessica A. Dominguez Rieg, Timo Rieg

**Affiliations:** 1 Department of Molecular Pharmacology and Physiology, Morsani College of Medicine, University of South Florida, Tampa, Florida, U.S.A.; 2 James A. Haley Veterans’ Hospital, Tampa, Florida, U.S.A.

**Keywords:** chronic kidney disease, fibroblast growth factors, heart failure, hypertension, phosphate, small molecules

## Abstract

Hyperphosphatemia results from an imbalance in phosphate (P_i_) homeostasis. In patients with and without reduced kidney function, hyperphosphatemia is associated with cardiovascular complications. The current mainstays in the management of hyperphosphatemia are oral P_i_ binder and dietary P_i_ restriction. Although these options are employed in patients with chronic kidney disease (CKD), they seem inadequate to correct elevated plasma P_i_ levels. In addition, a paradoxical increase in expression of intestinal P_i_ transporter and uptake may occur. Recently, studies in rodents targeting the renal Na^+^/P_i_ cotransporter 2a (Npt2a), responsible for ∼70% of P_i_ reabsorption, have been proposed as a potential treatment option. Two compounds (PF-06869206 and BAY-767) have been developed which are selective for Npt2a. These Npt2a inhibitors significantly increased urinary P_i_ excretion consequently lowering plasma P_i_ and PTH levels. Additionally, increases in urinary excretions of Na^+^, Cl^−^ and Ca^2+^ have been observed. Some of these results are also seen in models of reduced kidney function. Responses of FGF23, a phosphaturic hormone that has been linked to the development of left ventricular hypertrophy in CKD, are ambiguous. In this review, we discuss the recent advances on the role of Npt2a inhibition on P_i_ homeostasis as well as other pleiotropic effects observed with Npt2a inhibition.

## Introduction

Plasma phosphate (P_i_) is tightly controlled in the range from 0.9 to 1.45 mmol L^−1^ in healthy adults [1], and requires a concerted interplay between intestinal uptake, storage/release from bone, and renal excretion. The kidney plays an important role in fine-tuning total body P_i_ via modulating its reabsorption. Hyperphosphatemia is a common consequence of deranged P_i_ homeostasis and is classified as plasma P_i_ levels >1.45 mmol L^−1^ [1,2]. Hyperphosphatemia can further be subclassified as mild (1.44–1.76 mmol L^−1^), moderate (1.76–2.08 mmol L^−1^), or severe (>2.08 mmol L^−1^) [3].

In the kidney, P_i_ transport occurs mainly in the proximal tubule where the sodium–phosphate cotransporters Npt2a (SLC34A1), Npt2c (SLC34A3), Pit1 (SLC20A1), and Pit2 (SLC20A2) are expressed (for a review see [4–6]). The overall contribution of some of these transporters in P_i_ reabsorption has been elucidated by using genetically modified mice. The renal contribution of Npt2a has been estimated to be ∼70%, based on studies in brush border membrane vesicles of Npt2a^−/−^ mice [7], and is associated with increased urinary P_i_ excretion and reduced plasma P_i_ levels [8]. The contribution of Npt2c to P_i_ reabsorption is less clear. Kidney-specific Npt2c knockout does not affect P_i_ homeostasis [9] and Npt2a/c double knockout mice show similar urinary P_i_/creatinine ratios compared with Npt2a^−/−^ mice [10]; however, a compensatory up-regulation (∼2.5-fold) of Npt2c protein in brush border membrane vesicles of Npt2a^−/−^ mice was observed [11]. The contribution of Pit1 to renal P_i_ transport seems small and no differences in plasma P_i_ between Pit1^−/−^ and wild-type mice were found [12]. Knockout of Pit2 does not affect urinary P_i_/creatinine or plasma P_i_ when fed a control diet but high dietary P_i_ causes elevated plasma P_i_ compared with wild-type mice [13]. Of note, a recent study discovered that Npt2b, a transporter described to be primarily expressed in the intestine and lungs, was localized to the thick ascending limb; however, Npt2b was not regulated by dietary P_i_ [14].

The primary regulators of renal P_i_ excretion are PTH and FGF23, both mediating the retrieval of Npt2a/c from the luminal membrane (for a review see [15]). PTH is released from the parathyroid glands in response to low plasma Ca^2+^ levels as well as high plasma P_i_ levels [16]. The mechanism how FGF23 is released in response to elevated plasma P_i_ is less clear; however, it has been postulated that activation of FGF receptor 1 in response to elevated plasma P_i_ can regulate FGF23 production as well as via production of GALNT3, an O-glycosylation enzyme that protects FGF23 from proteolytic cleavage [17]. Even though elevated plasma P_i_ is considered a driver for secondary hyperparathyroidism and elevated plasma FGF23 levels, hyperphosphatemia does not occur until later stages in CKD. Plasma FGF23 starts to increase in the early stages of CKD which is followed by an increase in PTH. These changes are sufficient to maintain P_i_ levels in the ‘physiological' range until stage 4–5 CKD. This hypothesis is supported by studies showing that administration of a FGF23 neutralizing antibody increased plasma P_i_ in a rat model of reduced kidney function (5/6 Nx) [18], implying that elevated FGF23 levels are important for reducing plasma P_i_ levels in CKD. In addition, mice with drastically reduced FGF23 production in bone also showed increased plasma P_i_ levels in a mouse model of adenine-induced CKD [19]. Although elevated FGF23 levels are associated with a poor outcome in CKD (causing left ventricular hypertrophy [20]), FGF23 also protects the body from the detrimental cardio-renal consequences of elevated P_i_. Hyperphosphatemia alone is associated with cardiovascular morbidity and is also a substantial risk factor for the development of vascular calcification in CKD [21].

## Consequences of CKD on renal P_i_ transporters

How does CKD affect Npt2a expression? A continuous decline in nephron number in conjunction with elevated PTH and FGF23 levels potentially reduces Npt2a (and possibly Npt2c) expression. Consistent with this, in mouse and rat CKD models induced by adenine feeding, reduced expression levels of Npt2a protein and mRNA were observed [22–24]. In a different model of reduced kidney function in rats and mice (5/6 Nx), both species showed reduced Npt2a mRNA expression compared with sham animals [23,25]. Of note, renal Npt2b became highly expressed in the thick ascending limb in a model of oxalate-induced kidney failure [14]. Similarly, in adenine-induced CKD, renal Npt2b mRNA levels were ∼20-fold greater compared with control mice [23]. The significance of these findings remains to be determined but raises the question if increased renal Npt2b expression in CKD paradoxically increases P_i_ reabsorption. Another feature of CKD is low urine pH, and the activity of Npt2a increases with higher pH [26]. Some of these observations could impact the therapeutic efficacy when targeting renal Na^+^/P_i_ cotransporters.

## Therapeutic approaches to reduce P_i_ burden in CKD

Treating hyperphosphatemia in CKD remains a significant challenge. Currently, the treatment options for hyperphosphatemia are limited to dietary P_i_ restriction, oral P_i_ binders, and niacin/nicotinamide; however, all have been proven to have limitations [27–29]. The efficacy of dietary P_i_ restriction and P_i_ binders is further limited due to increased maladaptive P_i_ uptake in the gastrointestinal tract [30,31]. With the vision of inhibiting P_i_ uptake from the intestine, a non-absorbable Npt2b inhibitor (in the intestine Npt2b is responsible for >90% of active P_i_ uptake [32]) was developed [33]. Unfortunately, clinical trials with this compound were discontinued due to ineffectiveness in reducing plasma P_i_ in healthy volunteers and patients on hemodialysis [33]. A novel pan-phosphate transporter inhibitor (EOS789: Npt2a, Pit1/2) was able to maintain lower levels of plasma P_i_ in parallel with lower FGF23 and PTH in a long-term study [34] and was found to be safe in a phase 1b clinical trial in patients on hemodialysis [35]. Further studies are needed to prove its efficacy. Tenapanor, a non-absorbable intestinal-specific Na^+^/H^+^ exchanger isoform 3 inhibitor, was found to be an effective drug to reduce plasma P_i_ and FGF23 levels in patients on hemodialysis [36,37]. However, the United States Food and Drug Administration denied approval because of an effect that was ‘…small and of unclear clinical significance' [38], and requested additional studies before approval will be granted.

## Development of Npt2a inhibitors — *in vitro* studies

Until recently, no selective inhibitors were developed that target renal Npt2a. Pfizer developed PF-06869206 [39], which has a 3-chloro-2-methylazaindole core. Cell culture experiments in HEK cells, transfected with human, rat or mouse P_i_ transporters (Npt2a, Npt2c, Pit1 or Pit2), showed selectivity for Npt2a over all other tested transporters, with an IC_50_ ∼500 nmol L^−1^ [39]. An i.v. dose of 1 mg kg^−1^ in rats showed a half-life of 4.8 h, while a dose of 5 mg kg^−1^ in mice showed a half-life of 0.75 h. At higher oral doses, supra-proportional increases in plasma concentrations were observed, possibly indicative of saturation of clearance or enterohepatic recycling resulting in longer apparent half-lives. Bayer developed BAY-767 (structure not publicly available) with an IC_50_ of ∼4 nmol L^−1^ for rat/human Npt2a and >100-fold selectivity over Npt2b, Npt2c or Pit-1 in stably transfected CHO cells [40]. No data regarding half-life are available for this compound.

The inhibitory kinetics of PF-06869206 were also studied by our group in OK cells which endogenously express Npt2a, Npt2c, Pit1 and Pit2 and are a commonly used model to study electrolyte and P_i_ transport in the proximal tubule [41–43]. In OK cells, PF-06869206 caused a dose-dependent inhibition of Na^+^-dependent ^32^P_i_ uptake (IC_50_ ∼1 µmol L^−1^), reaching a maximum inhibitory effect of ∼70% at 100 µmol L^−1^. The nonselective Npt2 inhibitor PFA inhibited ∼90% of Na^+^-dependent P_i_ uptake, indicating that, in addition to Npt2a, Npt2c is inhibited [8]. Comparable results were found in acutely isolated rat proximal tubule cells: PF-06869206 inhibited ∼55% of ^32^P uptake at the maximum tested concentration of 30 µmol L^−1^ and PFA inhibited 70% of Na^+^-dependent P_i_ uptake [44]. Michaelis–Menten kinetics in OK cells identified no significant difference in apparent maximum velocity of reaction; however, showed a ∼2.4-fold higher substrate concentration for P_i_ in response to PF-06869206, which is consistent with a competitive mode of inhibition [8].

## *In vivo* effects of Npt2a inhibition — urinary P_i_ excretion

PF-06869206 shows good bioavailability in rats and mice. In our studies, acute oral gavage of PF-06869206 caused a dose-dependent increase in urinary P_i_ excretion in C57Bl/6J mice. The maximum dose of 100 mg kg^−1^ caused a ∼6-fold increase (3 h period) in urinary P_i_ excretion compared with vehicle (ED_50_ ∼21 mg kg^−1^) [45]. Studies looking at fractional excretion index (FEI) showed that FEI increased with higher doses of the inhibitor. At the highest dose tested (500 mg kg^−1^), a ∼17-fold increase in the FEI of P_i_ was observed (4 h period). BAY-767 caused a ∼1.7-fold increase in fractional urinary P_i_ excretion over a 16 h period at the highest dose tested (10 mg kg^−1^) [40]. Studies by 2 different groups confirmed the specificity of PF-06869206 by using Npt2a^−/−^ mice which completely lack a change in urinary P_i_ excretion [8,44]. In terms of the increase in urinary P_i_ excretion, Npt2c^−/−^ mice were indistinguishable from wild-type mice. Other models in which the phosphaturic effect of PF-06869206 has been tested are FGF23^−/−^ mice (characterized by growth retardation, abnormal bone phenotype, hyperphosphatemia and short life span [46]) and GALNT3^−/−^ mice (characterized by increased FGF23 proteolysis consequently lowering intact FGF23 levels and leading to hyperphosphatemic familial tumoral calcinosis [47]). In both models, FEI of P_i_ increased in response to PF-06869206 ∼9-fold and ∼2-fold, respectively, at a dose of 300 mg kg^−1^ compared with vehicle [44].

## Effects of Npt2a inhibition on plasma P_i_, PTH and FGF23

Do the above-described effects of Npt2a inhibition on urinary P_i_ excretion reduce plasma P_i_ levels and subsequently affect PTH and FGF23 levels? Our studies in C57BL/6J mice show that PF-06869206 at a dose of 30 mg kg^−1^ reduced plasma P_i_ starting after 1 h and reaching a maximum (−35%) 2 h after administration, with a full recovery after 24 h [8,45]. Somewhat unexplained is the 10-fold higher dose (300 mg kg^−1^) required to demonstrate a significant decrease in plasma P_i_ in C57BL/6 mice in the studies performed by Clerin et al. [44]. Two to four hours after administration of PF-06869206, plasma P_i_ significantly decreased in Npt2c^−/−^ (-33%), FGF23^−/−^ (-20%) and GALNT3^−/−^ mice (−20%) [44], the latter two providing evidence for efficacy of PF-06869206 under hyperphosphatemic conditions. Our unpublished observations also showed that PF-06869206 reduced plasma P_i_ levels in mice fed a high P_i_ diet. However, both studies [40,41] independently confirmed the specificity of these effects by using Npt2a^−/−^ mice. Three-day treatment of rats with BAY-767 resulted in ∼20% lower plasma P_i_ levels at 10 mg kg^−1^ [40].

The membrane abundance of Npt2a can be directly regulated by PTH and FGF23. Our studies in C57BL/6J mice showed a ∼50% reduction in PTH 3 h after administration of PF-06869206 at a dose of 30 mg kg^−1^. Clerin et al. [44] reported that in mice, PTH levels were ∼65% lower in response to 300 mg kg^−1^ PF-06869206 compared with vehicle 2 and 4 h after administration. Both studies indicated that 24 h after administration PTH levels returned to baseline. Rats treated with BAY-767 also showed ∼50% lower PTH after 3 days of treatment with 10 mg kg^−1^ compared with vehicle [40]. Of note, the Ca^2+^-sensing receptor in parathyroid glands also functions as a P_i_ sensing receptor explaining the stimulatory effect of P_i_ on PTH release [16]. In contrast with PTH, FGF23 levels were not significantly affected by PF-06869206 treatment in mice [44,45]. Of note, in rats treated with BAY-767, a ∼25% reduction in FGF23 was observed [40]. The reason for these discrepancies in FGF23 responses remains to be determined.

## Npt2a inhibition in CKD/reduced kidney function

**Figure 1.**
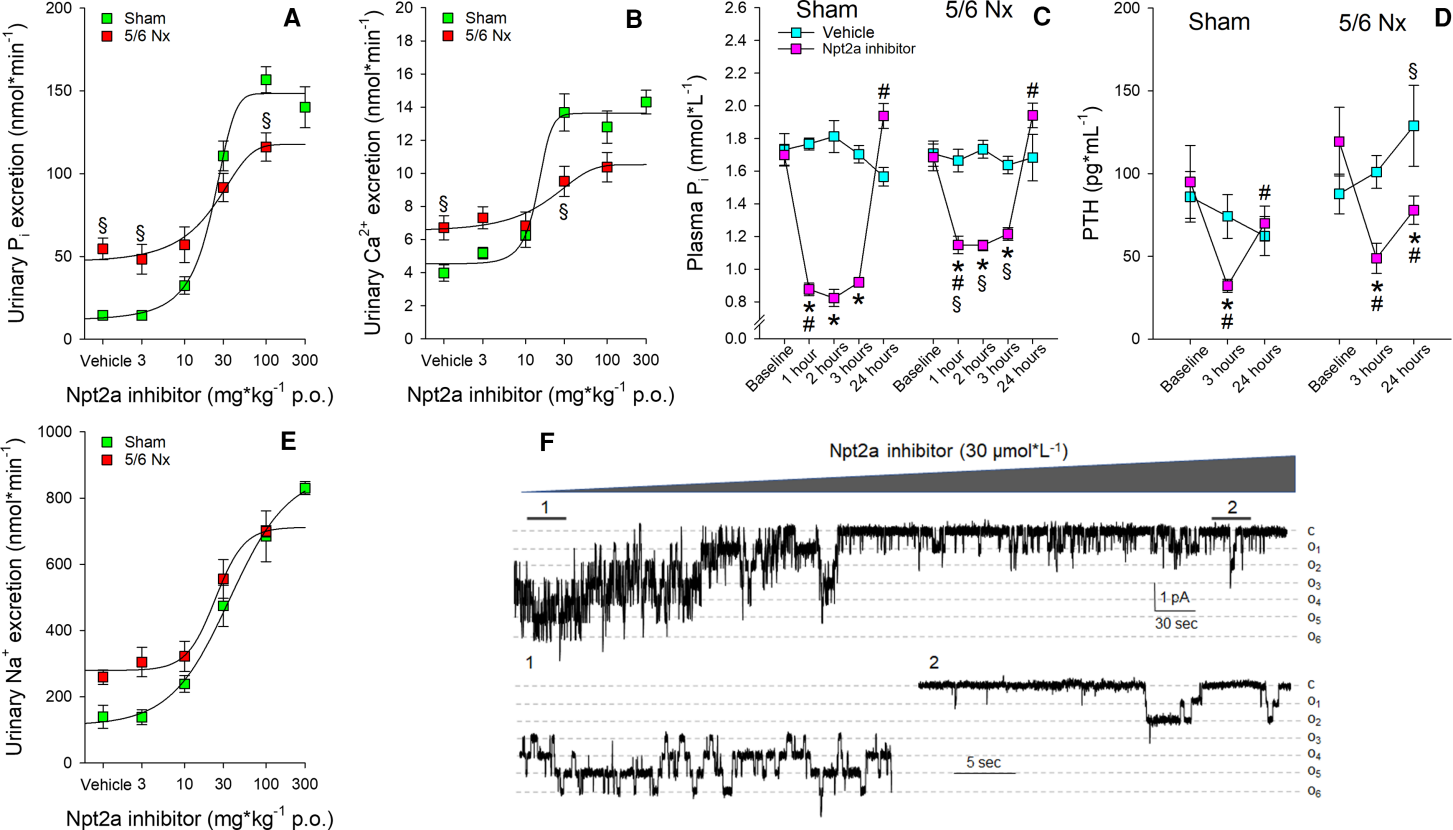
Pharmacological effects of Npt2a inhibition with PF-06869206 on renal and plasma parameters in mice with normal kidney function and 5/6 nephrectomy. Eight weeks after subtotal nephrectomy (5/6 Nx) or sham surgery, inhibition of Npt2a by PF-06869206 (given via oral gavage, p.o.) caused a dose-dependent increase in urinary (A) P_i_ and (B) Ca^2+^ excretion (3 h metabolic cage experiments). This was associated with reductions in (C) plasma P_i_ and (D) PTH levels in sham and 5/6 Nx mice, while plasma Ca^2+^ remained unaffected (not shown). At a dose of 100 mg kg^−1^, effects on plasma P_i_ were smaller in magnitude in 5/6 Nx compared with sham mice; however, the effect on plasma PTH was not different between groups. In addition to the effect of PF-06869206 on urinary P_i_ and Ca^2+^ excretion, a dose-dependent increase in urinary Na^+^ excretion (E) was found in both groups, that was not significantly different from each other. Due to a lack of effect on urinary K^+^ excretion and unaffected natriuresis in Npt2a^−/−^ mice (both not shown), we hypothesized that the natriuresis is a result of inhibition of Na^+^ transport in the connecting tubule/collecting duct, rather than in the proximal tubule, where the epithelial Na^+^ channel ENaC is expressed. In electrophysiological studies in acutely split-open cortical collecting ducts of C57BL/6 mice, ENaC open probability was measured in cell-attached patches formed on the apical membrane of principal cells. The pipette was backfilled with Npt2a inhibitor (30 µmol L^−1^). A continuous current trace is shown in (F). The areas under the bars over the continuous traces are shown below at expanded timescales. Dashed lines indicate the respective current levels, with *c* denoting the closed state and *o* denoting the open state. Open probability was acutely inhibited (∼85%) by PF-06869206, providing evidence that the natriuresis might be the consequence of off-target effects on ENaC. Data taken from [8,45]. *P < 0.05 versus vehicle, ^§^*P *< 0.05 versus sham, ^#^*P *< 0.05 versus previous time point.

As a model of reduced kidney function, our group employed 5/6 Nx in mice. However, in our studies, this model lacked some key features of CKD, such as hyperphosphatemia and secondary hyperparathyroidism. Eight weeks after surgery, acute administration of PF-06869206 showed a dose dependence for urinary P_i_ excretion (Figure 1A); however, the maximum effect (at 100 mg kg^−1^) was smaller in 5/6 Nx mice compared with sham mice (∼2-fold versus ∼10-fold, respectively). A dose of 100 mg kg^−1^ resulted in a significant decrease in plasma P_i_ (∼35% versus ∼50%, respectively; 1–3 h after administration, Figure 1C) and PTH (∼60% versus ∼65%, respectively, 3 h after administration, Figure 1D). Clerin et al. [44] studied 5/6 Nx rats that were treated with PF-06869206 (300 mg kg^−1^, q.d. via oral gavage) for 8 weeks. This long-term administration showed that FEI of P_i_ was ∼2.5-fold higher compared with vehicle treated 5/6 Nx rats. Plasma P_i_ was ∼15% lower in PF-06869206-treated compared with vehicle treated 5/6 Nx rats. In rats with 5/6 Nx, there was also no hyperphosphatemia, but secondary hyperparathyroidism and elevated FGF23 levels were observed compared with sham rats; however, treatment with PF-06869206 did not reduce PTH or FGF23 levels. So far, BAY-767 has not been tested in CKD models.

## Pleotropic effects of Npt2a inhibition

Are other minerals and electrolytes affected by Npt2a inhibition? In addition to effects on urinary P_i_ excretion, PF-06869206 increased urinary Na^+^, Cl^−^ and Ca^2+^ excretion (∼5-fold, ∼5-fold and ∼3-fold greater at 300 mg kg^−1^ compared with vehicle, respectively) in our studies without affecting their respective plasma levels [45]. Effects on urinary Ca^2+^ excretion (∼5-fold greater at 300 mg kg^−1^ compared with vehicle) have been confirmed by Clerin et al. [44], other parameters were not determined and are also not reported for BAY-767. The mechanism for the increased Ca^2+^ excretion remains elusive but could be caused by either inhibition of Ca^2+^ reabsorption in the proximal tubule or in the distal convoluted tubule where TRPV5 is expressed. The latter could be inhibited by decreased PTH in response to PF-06869206, leading to increased Ca^2+^ excretion. Of note, we did not observe effects on urinary K^+^, glucose or amino acid excretion, and urinary pH remained unaffected. Together this implies that there is no generalized effect on the proximal tubule as found, for example, in Fanconi syndrome. Similarly, in mice with 5/6 Nx, PF-06869206 caused dose-dependent responses in urinary Na^+^ (Figure 1E), Cl^−^ and Ca^2+^ (Figure 1B) excretion without affecting excretion of urinary K^+^, glucose, or pH [45].

We expected that the increase in urinary Na^+^ excretion would be blunted in Npt2a^−/−^ mice; however, to our surprise, the PF-06869206-induced natriuresis was still present in Npt2a^−/−^ mice [8]. In conjunction with the lack of effect on urinary K^+^ excretion, we hypothesized that this might be an off-target effect in the aldosterone-sensitive distal nephron via inhibition of the ENaC. Subsequent studies in acutely isolated, split-open cortical collecting ducts (Figure 1F) showed that ENaC open probability was reduced by ∼85% in response to PF-06869206 [8], giving a possible explanation why Npt2a^−/−^ mice still showed a natriuresis. Total body Na^+^ and blood pressure are interdependent variables; however, despite the acute natriuresis and diuresis observed in 5/6 Nx mice in response to PF-06869206 [45], long-term treatment with PF-06869206 in 5/6 Nx rats did not affect systolic blood pressure [44]. The reason(s) for these differences remain to be determined.

## Effect of Npt2a inhibition on vascular calcification

**Figure 2.**
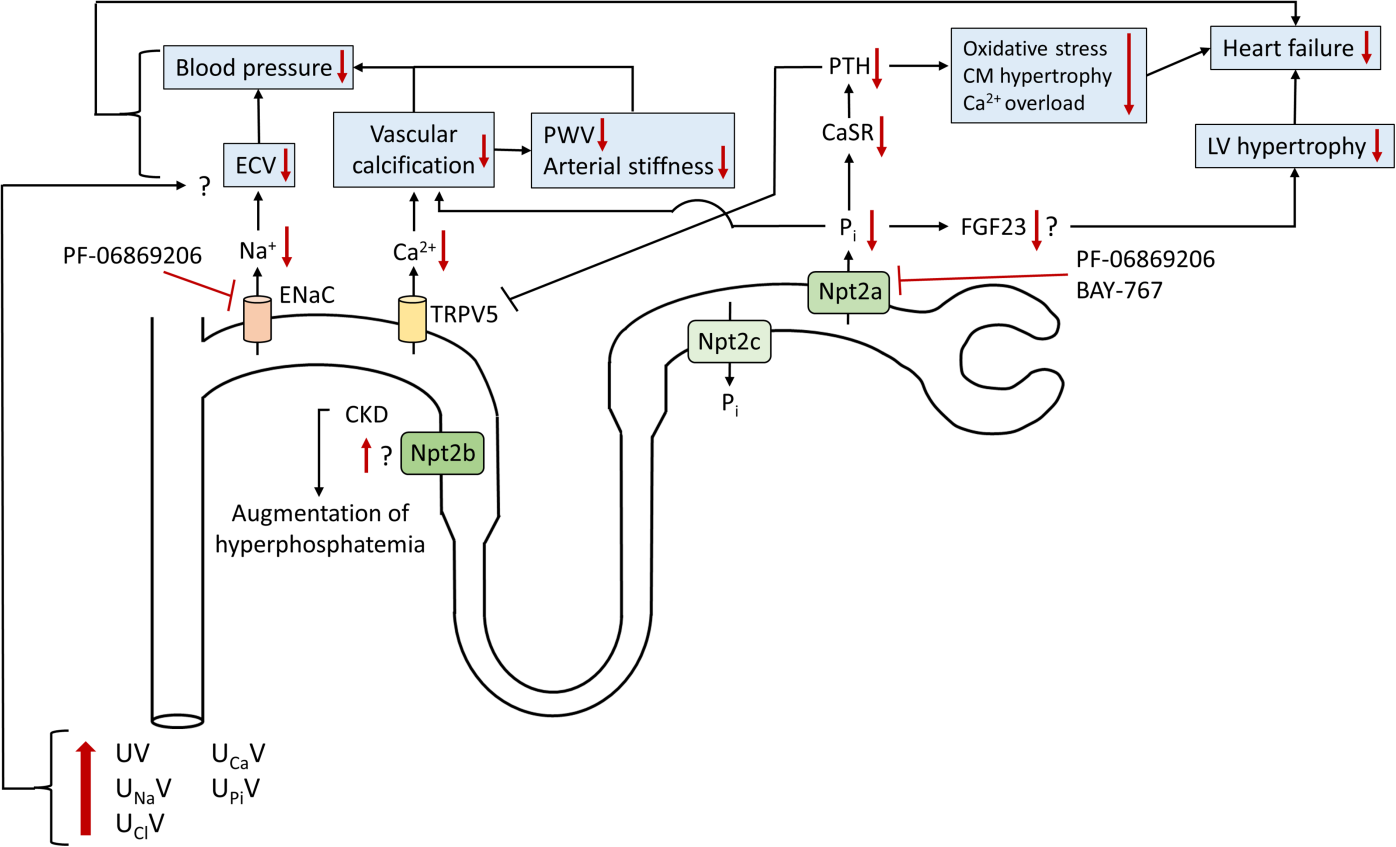
Proposed effects of Npt2a inhibition on renal electrolyte/mineral excretion and the potential role in cardiovascular protection. Npt2a blockade with either PF-06869206 or BAY-767 increases urinary P_i_ excretion and consequently, plasma P_i_ and PTH are reduced. The reduction in PTH, which most likely occurs via reduced activation of the calcium-sensing receptor (CaSR) on the parathyroid glands, may be protective against development of heart failure due to reduced PTH-induced cardiomyocyte (CM) hypertrophy, cardiac Ca^2+^ overload, and increased oxidative stress. Elevated FGF23 causes left ventricular (LV) hypertrophy in chronic kidney disease (CKD). Npt2a inhibition with BAY-767, but not PF-06869206, resulted in a decrease in plasma FGF23. Lowering FGF23 could reduce LV hypertrophy and possibly the development of heart failure. The diuretic and natriuretic effects of Npt2a blockade, the latter via reduced open probability of the epithelial sodium channel (ENaC), should reduce the effective circulating volume (ECV) and blood pressure. Either via a direct effect of Npt2a blockade in the proximal tubule or indirectly via a reduction in PTH and transient receptor potential cation channel 5 (TRPV5)-mediated Ca^2+^ reabsorption, urinary Ca^2+^ excretion is increased. The calciuretic effect, in combination with the phosphaturic effect, should reduce vascular calcification, pulse wave velocity (PWV), and arterial stiffness. This is expected to reduce blood pressure and further slowdown the progression of heart failure. New data have provided evidence of increased Npt2b expression in CKD; however, further studies are needed to determine its (patho)physiological relevance.

Another pleiotropic effect that needs special consideration relates to the increase in Ca^2+^ (and P_i_) excretion in response to PF-06869206 and BAY-767. Hormonal, mineral and other changes observed in CKD provide the perfect storm for accelerated vascular calcification. The latter goes along with reduced arterial elasticity, hypertension and augmented pulse-wave velocity (Figure 2). Together with elevated FGF23 levels this can result in left ventricular hypertrophy and ultimately heart failure (Figure 2), which is most exaggerated in patients on hemodialysis [48] and contributes substantially to cardiovascular mortality in CKD. BAY-767 has been studied in this regard [40]. Vascular calcification in rats was induced via administration of a pan-FGF receptor inhibitor for 10 days, which increased plasma P_i_ levels ∼2-fold. Concomitant oral treatment with BAY-767 (10 mg kg^−1^, q.d.), resulted in a reduced (∼1.4-fold) increase in plasma P_i_. Calcium content in the aorta was reduced by ∼75% in response to BAY-767 treatment. Of note, 2.2% lanthanum carbonate (administered via diet), an oral phosphate binder, did not affect aortic Ca^2+^ content [49].

## Perspectives

- Current treatment options for hyperphosphatemia in patients with or without reduced kidney function are insufficient. Inhibitors of renal Npt2a offer a novel treatment avenue to correct this condition and its detrimental consequences (vascular calcification, left ventricular hypertrophy, heart failure).
- Recent research has shown the feasibility of this approach in animal models of normal and reduced kidney function. Elevated PTH and FGF23 levels suppress renal Npt2a in CKD; however, PF-06869206 was still effective in reducing plasma P_i_ and PTH in CKD models.
- Further studies in different CKD models are needed to investigate the role of Npt2a inhibition on the course of CKD as well as possible reductions in cardiovascular complications.

## Abbreviations

CHO: Chinese hamster ovary
CKD: chronic kidney disaease
ED_50_: effective dose 50
ENaC: epithelial sodium channel
FEI: fractional excretion index
FGF23: fibroblast growth factor 23
GALNT3: Polypeptide N-Acetylgalactosaminyltransferase 3
HEK: human embryonic kidney
OK: opossum kidney
PFA: phosphonoformic acid
PTH: parathyroid hormone
q.d.: quaque die
TRPV5: transient receptor potential vanilloid 5
5/6 Nx: 5/6 nephrectomy
